# Low incidence of pneumonia in COPD patients treated with inhaled corticosteroids undergoing pulmonary rehabilitation

**DOI:** 10.1186/s12948-018-0090-y

**Published:** 2018-05-16

**Authors:** Erminia Ridolo, Cristoforo Incorvaia, Irene Martignago, Laura Pessina, Fulvio Lauretani, Luciano Loperfido, Gian Galeazzo Riario-Sforza, Annalisa Broglia, Bruna L. Gritti, Lorenzo Panella

**Affiliations:** 1 Department of Medicine and Surgery Clinical, University of Parma, Via Gramsci 14, 43126 Parma, Italy; 2 Cardiac/Pulmonary Rehabilitation, ASST Pini/CTO, Via Bignami 1, 20126 Milan, Italy; 3 Geriatric Department, AOU of Parma, via Gramsci 14, 43126 Parma, Italy; 4 Division of Internal Medicine, Division of Sub Acute Care, Sesto San Giovanni Hospital, ASST Nord Milano, Via Giacomo Matteotti, 83, 20099 Sesto San Giovanni, Italy; 5 Department of Rehabilitation and Functional Recovery, ASST Pini/CTO, Milan, Italy

**Keywords:** COPD, Inhaled corticosteroids, Pneumonia, Pulmonary rehabilitation

## Abstract

**Background:**

Based on meta-analyses results, it is currently acknowledged that there is an increased risk of pneumonia in patients with chronic obstructive pulmonary disease (COPD) undergoing inhaled corticosteroids (ICS) treatment. However, this is not found to be true in those with asthma. No data on this risk are available for COPD patients involved in pulmonary rehabilitation program (PR).

**Methods:**

For 1 year, we prospectively studied 2 cohorts of COPD patients—undergoing PR and not undergoing PR. The first group included 438 patients undergoing PR of which 353 were treated with ICS, and 85 were treated with bronchodilators only. The second group was comprised of 76 COPD patients who were treated with ICS, but not PR. The control group consisted of 49 ICS-treated patients with asthma. The diagnosis of pneumonia, when suspected, had to be confirmed with a chest x-ray.

**Results:**

Overall, 6 cases of pneumonia were diagnosed in the first study group: 5 ICS-treated patients and 1 patient treated only with bronchodilators. This corresponded to a rate of 1.41 and 1.17%, respectively, compared to a rate of 6.6% in COPD patients not treated with PR, which was significantly higher (p = 0.029) than that in the first study group. No case of pneumonia was registered among patients with asthma.

**Conclusions:**

These findings suggest that a significantly lower incidence of pneumonia is found in COPD patients treated with ICS and PR than in patients treated with ICS but not with PR. This observation deserves to be investigated in large populations of PR-treated COPD patients, possibly in multi-centric cohort studies.

## Background

According to the guidelines of chronic obstructive pulmonary disease (COPD) management, inhaled corticosteroids (ICS) should be prescribed only to patients with a severe or very severe disease (stage 3 or 4), as assessed by a forced expiratory volume in 1 s (FEV1) lower than 50% of the predicted value or by frequent (more than 2/year) exacerbations [1]. In recent years, an increasing number of reports disclosed that in COPD patients treated with ICS, there was a higher risk of developing pneumonia [2, 3]. Systematic reviews and meta-analyses confirmed the significantly higher risk of pneumonia in ICS-treated COPD patients compared with non-ICS treated patients; no significant effect on the mortality rate was detected [4–7].

In a meta-analysis, a 12-month budesonide treatment did not increase the risk of pneumonia [5]. According to a review, “the immunosuppressive effects of ICS on the respiratory epithelium and the disruption of the lung microbiome are most likely to be implicated”, and the conventional ICS doses are most likely too high [8]. In a UK cohort of 23,013 COPD patients, it was reported that there was a greater risk of pneumonia in patients receiving higher ICS doses [9]. In the same study, based on the lower risk of pneumonia observed in patients treated with extra-fine particle ICS compared with those treated with fine-particle ICS [9], it was suggested that the kind of the inhalant preparation administered could possibly influence the risk of pneumonia. Thus far, no such studies have been conducted on COPD patients treated with ICS and undergoing pulmonary rehabilitation (PR). This treatment consists of a multidisciplinary care program individually tailored and designed for patients with chronic respiratory impairment in order to optimize physical and social performance and autonomy [10]. PR was shown to reduce COPD exacerbations, often related to respiratory infections, by targeting risk factors such as physical inactivity, decreased exercise capacity and impaired physical functions [11]. This study, carried out in 2016, was conceived to evaluate the incidence of pneumonia in a cohort of COPD patients undergoing PR and treated with ICS or bronchodilators as compared to a control group not treated with PR.

## Methods

The study prospectively evaluated a cohort of COPD patients undergoing PR based on the insufficient control of the disease despite appropriate drug treatment [10]. To be admitted to the PR program, patients must have a FEV1 lower than 80% of the predicted, as measured by spirometry. Patients were divided in three groups: the first group included PR patients treated with ICS; the second group included PR patients treated only by bronchodilators; and, the third group included non-PR patients treated with ICS. For all COPD patients, the level of exercise capacity was measured by a 6-min walking test [12] A further group of ICS-treated patients with asthma and a low risk of pneumonia, as shown by a meta-analysis of available randomized trials [13], served as a control group.

In 2016, all PR patients underwent four or 6-month interval rehabilitative treatments depending on the severity of the disease. PR was performed as previously described [14]. The program, based on a schedule of 12 sessions within a 6-week period, involved exercise training on a cycle-ergometer or treadmill, upper-limb and trunk exercises, respiratory muscle training, and COPD education. Patients were instructed to contact the attending nurse in case of any important health-related events. When symptoms or signs of pneumonia were detected by the general practitioner or the ED physician, the diagnosis of pneumonia had to be confirmed with a chest x-ray.

### Statistical analysis

Differences in incidence of pneumonia were analyzed by the Chi squared test, a p value lower that 0.05 being regarded as positive.

## Results

The cohort study included 438 COPD patients undergoing PR, of which 353 were treated with ICS or ICS-LABA combinations, and 85 patients were treated only with bronchodilators. Of those patients treated with ICS, 246 received fluticasone/salmeterol; 64 beclomethasone/formoterol, 26 budesonide/formoterol, 8 fluticasone/vilanterol, 4 fluticasone, and 5 ciclesonide. The group of COPD patients not receiving PR included 76 subjects treated with ICS or ICS-LABA combinations of which 42 received fluticasone/salmeterol, 16 beclomethasone/formoterol, 11 budesonide/formoterol, 5 fluticasone/vilanterol, 1 beclomethasone/formoterol, and 1 fluticasone. The control group of patients with asthma included 49 subjects of which 13 received fluticasone/salmeterol, 12 beclomethasone/formoterol, 12 budesonide/formoterol, 6 fluticasone/vilanterol, 3 fluticasone/formoterol, 2 budesonide and 1 ciclesonide. The initial pharmacological regimen was maintained throughout the study. The patients treated with ICS only were given this prescription because the spirometric values were near to normality, but had frequent exacerbations. Table 1 shows the demographic characteristics of the various groups of patients as well as the mean values of FEV1 and the 6-min walking test. Overall, in 2016, 6 cases of pneumonia (only one requiring hospitalization) were diagnosed in the cohort of patients undergoing PR, 5 in ICS treated patients, and 1 in patients treated only with bronchodilators, corresponding to a rate of 1.41 and 1.17%, respectively. The difference in the rates was not significant. In particular, among the ICS-treated patients with pneumonia, 4 were treated with fluticasone/salmeterol, and 1 with budesonide/formoterol, also this difference being not significant. In the COPD group not receiving PR, 5 cases of pneumonia (none requiring hospitalization) were diagnosed, which corresponded to a rate of 6.6%. Of the 5 patients with pneumonia, 3 were treated with fluticasone/salmeterol, 1 with beclomethasone/formoterol and 1 with budesonide/formoterol. A significant difference (p = 0.029) was detected comparing the number of patients with pneumonia in the study group vs. the number of patients with pneumonia in the control group not receiving PR. No case of pneumonia was registered in the control group of patients with asthma.

**Table 1:** Demographic data and functional values from the different groups of patients

Group	Gender	Mean age (years)	Mean FEV1 value (% predicted)	Mean 6-min walking test
COPD patients undergoing PR	303 males, 135 females	73.6	56.1	353.9
COPD patients on PR treated with ICS	234 males, 119 females	74.7	52.7	327.3
COPD patients on PR treated only with bronchodilators	69 males, 16 females	72.9	59.6	380.7
COPD patients not on PR treated with ICS	47 males, 29 females	71.5	54.2	358.4
Asthmatic patients treated with ICS	28 males, 21 females	54.7	71.5	N.A.

## Discussion and conclusions

**Fig. 1:**
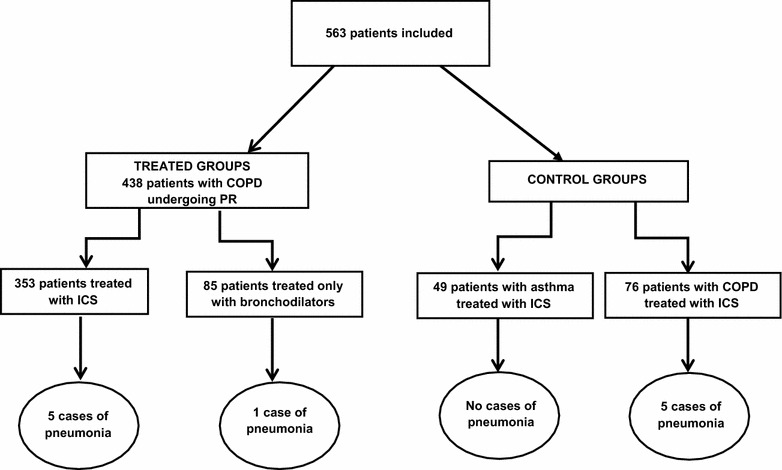
Study flowchart and grouping. *COPD* chronic obstructive pulmonary disease, *ICS* inhaled corticosteroids, *PR* pulmonary rehabilitation

There is clear evidence in the literature that the use of ICS in COPD patients increases the risk of pneumonia [4–7]. Based on this observation, the updated GOLD Guidelines recommend that ICS be prescribed only to patients with severe or very severe (GOLD 3 or 4) disease [15]. When the treatment is required, individuating the minimal effective dose of ICS in these patients will likely lessen the risk of pneumonia [16]. Our study suggests that PR may substantially reduce such risk (Fig. 1). In fact, our findings show that in ICS treated-patients undergoing PR the incidence of pneumonia was 1.41%. There was no significant difference in PR patients treated with bronchodilators compared to patients treated with ICS, instead the difference in rate was found to be significant in the cohort of patients treated with ICS but not with PR. We also compared the reported incidence of pneumonia in COPD patients, treated with or without ICS, and in the general population. The figures for COPD patients were obtained from the Cochrane meta-analysis of the risk of pneumonia stemming from ICS [6], while those concerning the general population were taken from the literature review from Torres et al. [17]. The available data are summarized in Table 2. It is evident that the incidence of pneumonia in COPD patients treated with ICS undergoing PR is significantly higher than the general population (p < 0.001), and significantly lower than COPD patients treated with fluticasone (p < 0.001). Our data do not confirm the lower incidence of pneumonia in patients treated with budesonide compared with those treated with fluticasone, as reported in meta-analyses [4, 5] and population studies [18], but the very low number of pneumonia in our study (5 and 1, respectively) cannot provide statistical reliability. The low number of pneumonia cases in both the study and control groups, related to the limited number of patients (< 100), affected the comparison of pneumonia incidences according to the different ICS used.

**Table 2:** Pneumonia events in the general population, in COPD patients and in COPD patients treated with ICS

General population Torres et al. [17]	COPD patients Kew et al. [6]	COPD patients treated with fluticasone Kew et al. [6]	COPD patients treated with budesonide Kew et al. [6]
1.07–4.2 cases per 1000 person-year. In Italy 1.7 cases per 1000 person-year	Controls in trials with fluticasone (follow-up 22 months): 72 per 1000 Controls in trials with budesonide (follow-up 10 months): 28 per 1000	Follow-up 22 months: 116 per 1000	Follow-up 10 months: 31 per 1000

According to a meta-analysis, PR has several potential benefits, e.g., relieving dyspnea and fatigue, improving exercise capacity, and health-related quality of life, thus enhancing the sense of control that individuals have over their condition. The authors stated that additional RCTs comparing pulmonary rehabilitation and conventional care in COPD are not warranted [19]. Instead, studies on large populations of PR-treated patients are necessary to confirm our observation of reducing the risk of pneumonia by using ICS. On the other hand, a favorable clinical course was reported in hospitalized patients with severe COPD undergoing PR treatment affected by acquired pneumonia while in hospital (HAP) [20]. The zero mortality rate mentioned is relevant, considering the substantial mortality due to HAP. The underlying factors of these outcomes of PR on pneumonia incidence could be possibly due to the beneficial effects of physical activity and patient education. Indeed, in a study on 441 adults undergoing upper abdominal surgery, patients were randomized to receive pre-operative physiotherapy and education (study group) or simply an information booklet (control group). The incidence of HAP was halved in the study group compared with the control group, with an absolute risk reduction of 15% [21].

Also, the risk of pneumonia is affected by the extent of the subjects physical activity. In fact, in a large population of 109,352 runners and 40,798 walkers, higher doses of running and walking (with no difference between the two) were associated with a lower risk of pneumonia, and a mortality rate decrease of 13%. This outcome appeared to be independent of the effect of exercise on cardiovascular diseases [22]. However, studies are needed to specifically address the incidence of pneumonia in active COPD patients according to the type and extent of their physical exercise. The absence of the risk of pneumonia in patients with asthma, which has been demonstrated by a meta-analysis [13], although a significant reduction in physical activity has been reported in patients with severe asthma [23], warrants further investigation.

## Abbreviations

COPD: chronic obstructive pulmonary disease
FEV1: forced expiratory volume in 1 s
HAP: hospital acquired pneumonia
ICS: inhaled corticosteroids
PR: pulmonary rehabilitation
